# The Evidence for Intravenous Theophylline Levels between 10-20mg/L in Children Suffering an Acute Exacerbation of Asthma: A Systematic Review

**DOI:** 10.1371/journal.pone.0153877

**Published:** 2016-04-20

**Authors:** Lewis Cooney, Daniel Hawcutt, Ian Sinha

**Affiliations:** 1 Department of Women's and Children's Health, Institute of Translational Medicine, University of Liverpool, Liverpool, Merseyside, United Kingdom; 2 National Institute for Health Research Alder Hey Clinical Research Facility, Alder Hey Children’s Hospital, Liverpool, Merseyside, United Kingdom; Cardiff University, UNITED KINGDOM

## Abstract

**Background:**

Intravenous theophyllines are a second line treatment for children suffering an acute exacerbation of asthma. Various guidelines and formularies recommend aiming for serum theophylline levels between 10-20mg/l. This review aims to assess the evidence underpinning this recommendation.

**Methods:**

A systematic review comparing outcomes of children who achieved serum theophylline concentrations between 10-20mg/l with those who did not. Primary outcomes were time until resolution of symptoms, mortality and need for mechanical ventilation. Secondary outcomes were date until discharge criteria are met, actual discharge, adverse effects and FEV1.

**Data sources:**

MEDLINE, CINAHL, CENTRAL and Web of Science. Search performed in October 2015.

**Eligibility criteria:**

Interventional or observational studies utilizing intravenous theophyllines for an acute exacerbation of asthma in children where serum theophylline levels and clinical outcomes were measured.

**Findings:**

10 RCTs and 2 observational studies were included. Children with serum levels between 10-20mg/l did not have a reduction in duration of symptoms, length of hospital stay or need for mechanical ventilation or better spirometric results compared with levels <10mg/l. Levels above 20mg/l are not associated with higher rates of adverse effects. This study is limited due to heterogeneity in the way theophylline levels were reported and poor surveillance of adverse effects across studies.

**Conclusion:**

Dosing strategies aiming for levels between 10-20mg/l are not associated with better outcomes. Clinicians should rely on clinical outcomes and not serum levels when using intravenous theophyllines in children suffering an acute exacerbation of asthma.

## Introduction

Asthma is a disorder of widespread lower airway inflammation and obstruction that is reversible either spontaneously or with treatment. Asthma affects around 235 million people worldwide and is a common cause of hospital admission in children [1]. In an acute exacerbation, inhaled medication may fail to control symptoms, resulting in potentially life threatening airways obstruction [2]. Intravenous theophyllines can be used as second-line therapy for children who do not respond to inhaled bronchodilators and systemic corticosteroids [3]. Aminophylline is a mixture of theophylline, which is the active compound that causes bronchodilation by poorly understood mechanisms [4], and ethyldiamine, an excipient which confers greater solubility in water. Intravenous preparations of theophylline have also been developed with different excipients to minimize potential allergic reactions.

It is advised that serum levels of theophylline should be measured, as it is purported to have a narrow therapeutic range, and its pharmacokinetic properties vary between patients [5,6]. The most recent guidelines and formularies recommend a therapeutic range between 10-20mg/l and a loading dose of 5mg/kg for children who do not take oral theophylline regularly [3,7]. This therapeutic range appears to have been originally based on studies demonstrating improvements in spirometry in adults with theophylline levels above 10mg/l [8–10]. As pharmacological properties of drugs change with age, this may not necessarily be appropriate in children. Furthermore, aminophylline is used in children with severe asthma attacks to prevent deterioration and resolve clinical symptoms, rather than improve physiological measures of lung function. We aimed to appraise the evidence for the current therapeutic range of aminophylline in children with acute asthma (10–20 mg/l).

## Materials and Methods

### Study design

We conducted a systematic review (S1 File) of studies investigating the use of intravenous theophyllines in acute asthma in children that report both relevant clinical outcomes and theophylline levels.

### Methods of the review

We included parallel and crossover randomized controlled trials (RCTs) comparing two or more therapeutic ranges for intravenous theophyllines in children and adolescents (aged 19 or younger) with acute asthma. We also included RCTs comparing intravenous theophyllines with placebo, if a measure of serum theophylline levels was reported for the two treatment groups, and we included retrospective or prospective observational studies if they reported results for both clinical outcomes and therapeutic levels measured in the included children.

We excluded studies including adults (20 years and older) and children unless the paediatric data were reported separately, and studies utilizing theophyllines for an indication other than asthma (e.g. neonatal apnoea and tuberculosis).

### Outcomes

The prespecified primary outcomes were time until resolution of symptoms, need for mechanical ventilation, and mortality [11]. Secondary outcomes were days until discharge criteria are met, actual discharge from hospital, adverse effects as defined and reported by authors, and forced expiratory volume in one second (FEV_1_). Studies must report at least one outcome to meet our inclusion criteria.

#### Identification of studies

The following search strategy was used to search MEDLINE, CINAHL, The Cochrane Central Register of Controlled Trials, and Web of Science in September 2015, with no date or language restrictions: ((aminophylline OR xanthine OR phyllocontin OR theophylline OR pde4 inhibitor OR phosphodiesterase 4 inhibitor OR caffeine) and (intravenous OR IV OR parenteral) AND (acute asthma OR asthmaticus OR severe asthma OR "hospital*ed" OR asthma attack") AND (child* OR adolescen* OR infan* OR p*ediatric))

One reviewer (LC) screened all abstracts. A second reviewer (IS or DH) checked the eligibility of abstracts after initial screening, and full studies included in the review. Reference lists were screened for other eligible studies.

### Data extraction and analysis

From each study we identified the theophylline levels achieved in the research participants (and when these were measured) and, if stated, the desired target range. We extracted data around the outcomes listed above, at whichever timepoints they were reported. We also recorded the age range of participants. From RCTs, exclusion criteria, control medication, concomitant medication and statistical significance of results were also noted.

We anticipated methodological and reporting heterogeneity between studies so we planned a priori that the results of the review would be presented descriptively.

The primary analysis was of RCTs comparing therapeutic ranges of theophylline, in which we aimed to compare clinical outcomes between groups. The secondary analyses were of RCTs comparing theophylline with placebo, and observational studies. In these studies, we tabulated results for our prespecified clinical outcomes (for RCTs, we recorded the magnitude of difference in outcome between theophylline and placebo), alongside either the mean or median serum theophylline level measured in the group, or the proportion of participants whose levels were within a predefined target therapeutic range. We assessed whether those RCTs in which mean or median theophylline levels were >10 mg/l reported more benefit than those in which the measured level was <10 mg/l, and whether those studies reporting levels >20 mg/l appeared to demonstrate a higher incidence of adverse effects.

Data was extracted by primary investigator LC and reviewed by DH. Any disagreements regarding the extraction process were resolved by consensus, or arbitration by reviewer IS.

### Assessment of quality of included studies

The Cochrane Risk of Bias Tool [12] was applied to each RCT, to help determine the validity of results. The critical appraisal skills programme (CASP) tool was used to appraise the quality of observational studies. We also evaluated whether authors described how they monitored individual children for adverse effects, and how thorough this surveillance was [13].

## Results

**Fig 1:**
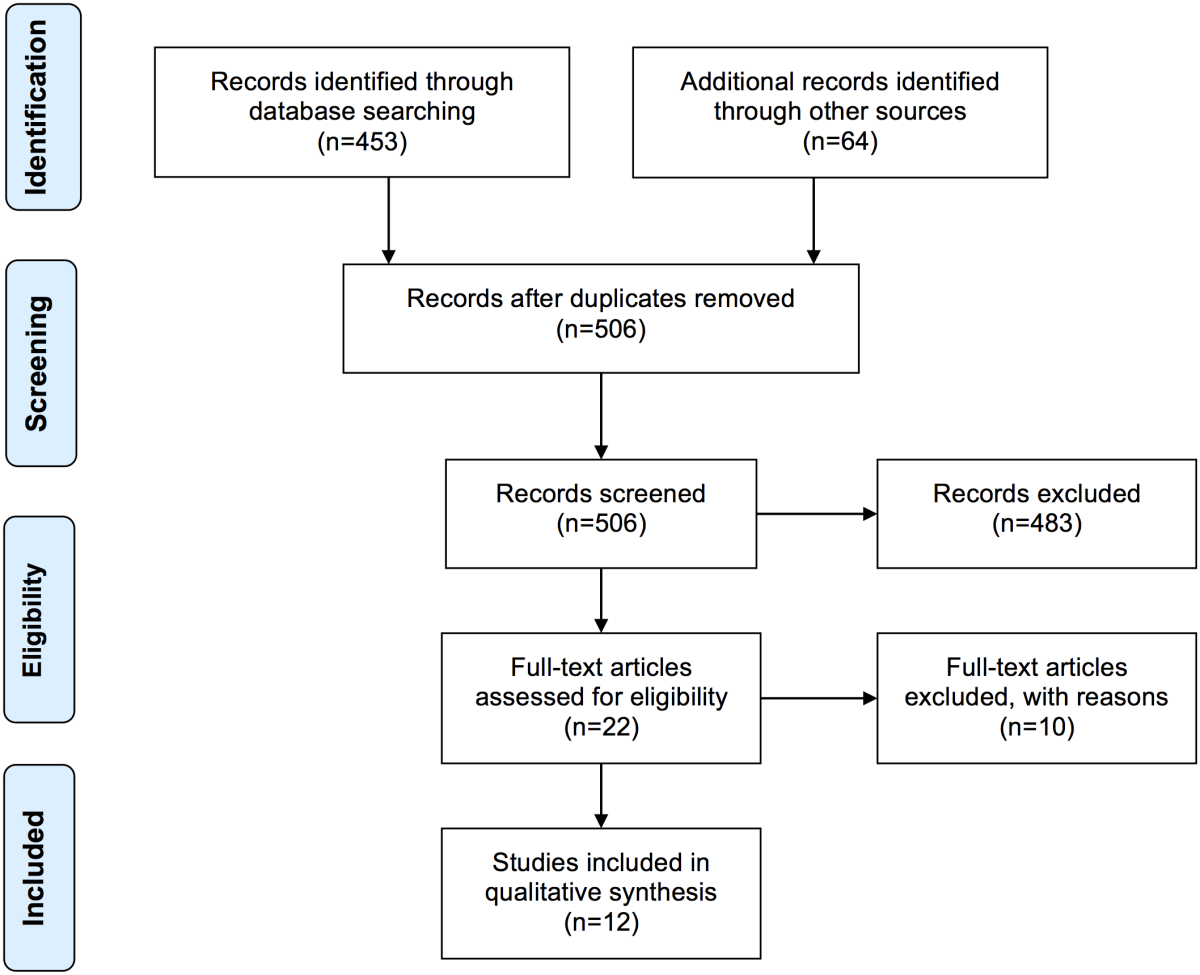
Search results.

A total of 488 studies were found using the search criteria, with 22 full text articles screened for eligibility. We excluded 10 full text articles (S2 File) with the remaining 12 articles included in the review (Fig 1).

We found no RCTs comparing different therapeutic ranges of theophylline. We included ten RCTs comparing theophylline with placebo, and two retrospective observational studies.

### Quality of included studies

Of the ten randomized controlled trials, two gave no data on adverse effects [14,15], 2 reported side effects unsystematically [16,17] and six reported adverse effects thoroughly using prospective methods clearly outlined in the methodology [18–23].

The results of the Cochrane risk of bias assessments conducted on the ten RCTs comparing intravenous theophyllines with placebo are shown in Table 1. Three studies were classed at high risk of attrition bias, one at high risk of reporting bias with respect to symptom scores, and three at high risk of reporting bias of adverse outcomes. In general, the other domains of bias were classified as low or unclear risk in most studies.

**Table 1:** Assessment of risk of bias in included studies using Cochrane tool.

	D’Áliva 2008	Ream 2001	Nuhoglu 1998	Yung 1998	Bien 1995	Needleman 1995	Strauss 1994	Carter 1993	DiGiulio 1993	Pierson 1971
**Random sequence generation (selection bias)**	**?**	↓	**?**	↓	↓	**?**	**?**	**?**	**?**	**?**
**Allocation concealment (selection bias)**	↓	↑	**?**	↓	**?**	↓	**?**	**?**	**?**	↓
**Blinding of participants (performance bias)**	↓	↑	↓	↓	↓	↑	**?**	↑	↓	↓
**Blinding of outcome assessment (performance bias)**	↓	↓	↓	↓	**?**	↓	↓	**?**	**?**	↓
**Incomplete outcomes assessed (attrition bias)**	**?**	↑	↓	↓	↓	↑	↑	↓	↓	**?**
**Selective outcome reporting (symptom scores/spirometry)**	↓	↓	↓	↓	↓	↓	↓	↓	↓	↑
**Selective outcome reporting (adverse reactions**	↓	↓	↑	↓	↓	↑	↓	↓	↑	↑

↓ low risk of bias, ↑ high risk of bias, ? unclear risk of bias

The CASP assessments conducted on the two observational studies are presented in Table 2

**Table 2:** Assessment of quality in observational studies.

Retrospective studies	Dalabih 2014	Fox 1982
Clearly focused issue	↓	↓
Acceptable recruitment	↓	↓
Adequate exposure measurement	↓	↓
Adequate outcome measurement	↓	↓
confounding factors identified	↑	↑
Complete follow up?	↑	↑
Result precision	**?**	**?**
Believable results	↓	↓
Applicable to local population	↓	↓
Consistent with other evidence	**?**	↓

↓ low risk of bias, ↑ high risk of bias, **?** unclear risk of bias

### Clinical outcomes

The theophylline levels reached, primary outcomes and secondary outcomes are shown in Tables 3 and 4.

**Table 3:** Results of Randomised Controlled Trials.

						Primary outcomes		Secondary outcomes			
First author, Location, age range of participants	Study design	Timing of theophylline level measurement	Theophylline levels achieved	Exclusion criteria	Other medication used	Time until resolution of symptoms	Need for mechanical ventilation	Date until discharge criteria are met	Actual discharge	Adverse effects	Spirometry
**D'Ávila 2008, Brazil, 2–5 years**	RCT comparing a bolus of aminophylline given twice to a bolus of saline given twice	Not measured	7.37mgl ±1.39mg/l (mean±SEM)	Mechanical ventilation prior to randomisation, xanthine allergy, seizure in the past week	β_2_ agonists, steroids	Not measured	Excluded	Not measured	No significant difference in length of hospital stay 30.8h in aminophylline group vs. 40.0h in placebo (p = 0.48)	Not measured	Not measured
**Pierson 1971, USA, 5–17 years**	RCT comparing aminophylline bolus and infusion with normal saline	24 hours after hospital admission	5-15mg/l	No exclusion criteria in methodology	Adrenaline	Not measured	Not measured	Not measured	Not measured	Not measured	Significantly improved FEV1 and FVC at 24 hours compared with placebo (89% vs 62% p<0.001)(18% vs 68% p<0.001)
**Ream 2001, USA, 13–17 years**	RCT comparing aminophylline bolus and infusion with normal saline	30 to 60 minutes after the loading dose and 4 to 6 hours after beginning the infusion	11.2±0.4mg/l after loading dose, 12.5±1.2 mg/L at 8 to 12 h average daily level of 14.5 ± 0.7 mg/L.	Xanthine allergy, STL>3mg/l, cardiovascular disease, pregnancy	β_2_ agonists, ipratropium bromide, steroids, terbutaline	Those receiving theophylline achieved a CAS of <3 sooner than control subjects (18.6±2.7 h vs 31.1±4.5 h; p<0.05)	All subjects intubated before infusion	Time to discharge criteria in theophylline group 29.8±4.9 hours vs. 36.4±5.5 hours in control p>0.05 In those not receiving mechanical ventilation. In subjects receiving mechanical ventilation 74.8±8.9 in theophylline group vs. 189.3±34.3 p<0.05	Length of stay in critical care in theophylline (3.9±0.3 days) vs. control (8.8±1.5 days) p<0.05, length of stay in hospital 8.3±1.5 days vs. 13.0±1.0 days p<0.06	High rate of adverse effects, no data on how these correlate to serum levels, did not differ between controls	Not measured
**Bien 1995, USA, 2–10 years**	RCT comparing theophylline bolus and infusion with normal saline	30 mins after loading dose, 6 hours later, once a day at therapeutic steady state level	10.1mg/l (mean) post bolus, 11.8(mean) at approximately 8 hours	Mechanical ventilation prior to randomisation, xanthine allergy, pneumonia, febrile, use of systemic steroids, STL>2.3mg/l	β_2_ agonists	CAS at 24 hours 2.0 in theophylline group 2.6 in placebo group p>0.05	Excluded	Not recorded	Not measured	Statically higher rates of nausea, and vomiting in theophylline group p≤0.05 but not insomnia p = 0.08	Peak flow only available in 5 patients, statistical analysis not possible
**Strauss 1994, USA, 5–18 years**	RCT comparing theophylline bolus and infusion with normal saline	30 minutes and 4 hours after the initial dose and then approximately every 12 hours.	Mean theophylline level 12.0±2.5mg/l mean of peak 14.3mg/l	Mechanical ventilation prior to randomisation, Wood Downes score>5, Serum theophylline >5mg/l, theophylline within the past 4 hours, drugs interfering with theophylline metabolism	β_2_ agonists, steroids	Not measured	Excluded	Not measured	Hospital stay in aminophylline group 2.58±1.5 days vs 2.33±1.3 days in placebo group p>0.2	Significantly higher rates of side effects in aminophylline group (43%) in vs control (6%) in p<0.052 patients removed due to toxicity, headache and abdominal pain, one patient theophylline level of 23mg/l and experienced nausea and vomiting, all other patients had levels >20mg/l.	PPFR final 0.80±0.22 in aminophylline group vs 0.79±0.22 in placebo p>0.2
**DiGiulio 1993, USA, 2–18 years**	RCT comparing aminophylline bolus and infusion with placebo	1 hour after starting infusion	13.1±3.4mg/l (mean) throughout study	Mechanical ventilation prior to randomisation, steroids within the past 2 weeks, pregnancy	β_2_ agonists, steroids	30.4±16.8 in intervention vs 27.0±10.3 hours in control; p = 0.51	Excluded	30.4±16.8 hours in aminophylline vs. 27.0±10.3 hours in control; p = 0.51. Discharge criteria was equated to CAS≤2	Not measured	Not statistically significant compared with control group	Not measured
**Nuhoglu 1998, Turkey, 2–16 years**	RCT comparing aminophylline bolus and infusion with normal saline	Within one hour of the completion of the loading dose, and 12–18 hours later	10.5–14.3mg/l throughout study	Theophylline administration in the past 48 hours	β_2_ agonists, steroids	CAS at 24 hours 2.1 placebo and 2.0 in aminophylline p = 0.8452	Not measured	Not measured	Not measured	No significant difference between groups. 2 patients with adverse effects were documented with therapeutic serum theophylline levels. No significant difference from control.	Only 2 children were able to perform spirometry, statistical analysis not possible
**Carter 1993, USA, 5–18 years**	RCT comparing theophylline bolus and infusion with normal saline	6, 12 to 24, and then every 24 hours thereafter	10-20mg/L in all patients throughout study	Hypercapnia, inability to perform spirometry	β_2_ agonists, steroids	Median CAS/PI at 36 hours 2 in intervention and control groups p = 1.0	Excluded	Not measured	Not measured	no clinically relevant adverse effects, no data on how these correlate to serum levels, did not differ significantly between controls	No significant difference in FEV1 at any time in the study p>0.05
**Needleman 1995, USA,2–18 years**	RCT comparing aminophylline bolus and infusion with normal saline	30 mins and 60 mins after initial bolus, 4 to 6 hours later on steady state infusion	10-20mg/L in all patients throughout study	Mechanical ventilation prior to randomisation, STL>2.5mg/l, theophylline in the past 48 hours, cardiac disease	β_2_ agonists, steroids	Fall in asthma score in treatment group 3.05±3.25 vs. 2.38±2.19 in placebo p = 0.482	Excluded	Not measured	Length of stay in theophylline group 52.3±32.3 hours vs. 48.2±26.6 hours p = 0.654	Not measured	Not measured
**Yung 1998, Australia, 1–19 years**	RCT comparing theophylline bolus and infusion with normal saline	12–18 hours post bolus	<10mg/l in 4(5%), 10–14.5 in 26(33%) 14.5–20 in 42(53%) and >20 in seven(9%) post loading dose, three (7%), 15 (35%), 11 (26%), and 13 (31%),	Pregnancy, other chronic respiratory disease, significant disease of other organ systems, previous theophylline within the past 48 hours	β_2_ agonists, ipratropium bromide, steroids	Not measured	All subjects intubated before infusion	Not measured	2.87 days in aminophylline group vs. 2.69 days in placebo p = 0.53	Statistically significantly higher rates of Nausea, vomiting in aminophylline group p = 0.05, no statistically significant differences in headache, irritability, tremour or seizures Patients on aminophylline were more likely to have their infusions stopped because of adverse effects	FEV1 @ 24 hours 22.5 in aminophylline vs 13.1 in placebo p = 0.029

RCT—Randomised controlled trial, CAS/PI—Clinical asthma score/pulmonary index, ASS—asthma severity score, RDS—respiratory distress score

**Table 4:** Results of Observational studies.

				Primary outcomes		Secondary outcomes			
First author, Location, age range of participants	Study design	Timing of theophylline level measurement	Theophylline levels achieved	Time until resolution of symptoms	Need for mechanical ventilation	Date until discharge criteria are met	Actual discharge	Adverse effects	Spirometry
**Fox 1982, USA,1–16 years**	Retrospective analysis of patients' theophylline levels on subsequent therapeutic decisions	Not measured	<10mg/l in 20 patients 10-20mg/l in 14 patients, no patients had levels >20mg/l	Not measured	Not measured	Not measured	3.25 days	3 patients with theophylline levels 20.5mg/l, 21.1mg/l and 25.6mg/l non showed signs of theophylline toxicity	Not measured
**Dalabih 2014, USA, 3–18 years**	Retrospective analysis of critical care patients admitted with an acute exacerbation of asthma were compared with similar patients who did not	Not measured	31 had theophylline levles≥10mg/l, 18 had theophylline levels≤10mg/l	Time to reach RDS*≤7 longer in those who received aminophylline compared to those who did not (HR = 0.359 95% CI [0.223, 0.578] p<0.001. Longer in those with levels 10-20mg/l HR = 0.403 CI [0.204, 0.739] p = 0.008	Not measured	Not measured	Aminophylline associated with longer stay in critical care HR = 0.396 CI[0.245, 0.64] p<0.001. Among those who receive aminophylline length of stay was longer HR = 0.457 CI [0.234, 0.895] p = 0.023	Not measured	Not measured

RDS—Respritory distress score, HR—hazard ratio, CI—confidence interval

### Clinical outcomes in context of theophylline levels

Of 10 RCTs six gave an optimal therapeutic range for theophylline: three studies aimed for serum concentrations between 10 and 20mg/l [14,19,21] one study of 15mg/l [23], one between 12-17mg/l [18] and one between 12-20mg/l [16]. Of the observational studies one defined target therapeutic levels as 10mg/l or greater [24] and one as 10-20mg/l [25].

There was non uniformity in the timing of theophylline level measurement. Of the randomized controlled trials, three measured serum levels 30 minutes after completion of the loading dose [14,22,23], three after one hour of completion of the loading dose [18–20], and one six hours after completion of loading dose [16]. One RCT and neither observational study did not stipulate when theophylline were taken [5,17,21]. Serum levels were measured in all participants receiving theophylline, except in one study, where only 17% of those in the intervention group had serum theophylline levels measured [15].

There was heterogeneity between studies in the way in which theophylline levels were reported. Five studies presented the mean theophylline level achieved [16,16,81,21,22], three gave the proportion of research participants who were below/above the target range [20–24, 25] and four gave the range of theophylline levels achieved [14,17,19,23].

### Primary Outcomes

#### 1) Time until resolution of symptoms

Of the ten RCTs, two reported time until resolution of symptoms as an outcome [18–26], four measured change in asthma score over a given time [14,19,21,23] and four did not measure symptoms. Various symptom scores were used (Table 4).

There appeared to be no difference in the magnitude of results when comparing levels of serum theophylline measured in participants. In one RCT demonstrated that symptom improvement was quicker in those receiving theophylline compared with placebo (18.6±2.7h vs 31.1±4.5h [p<0.05], mean serum theophylline levels 11.2mg/l) [18] but this was not replicated in another study in which similar serum theophylline levels were reported (30.4±16.8h vs 27.0±10.3h [p = 0.51], mean serum theophylline level 13.1mg/l) [16] No studies demonstrated a statistically significant improvement of symptoms after 2, 6, 12, 24, 48 and 36 hours at any serum theophylline level [14, 19, 21–23].

One retrospective study [24] measured time until symptom improvement and found that this appeared to be longer after treatment with aminophylline (hazard ratio 0.359, p < 0.001). The authors also note that this was significantly more prolonged in those with levels >10mg/l compared to those who are subtherapeutic (hazard ratio 0.403 p = 0.0085).

#### 2) Need for mechanical ventilation

No studies compared the effect of IV aminophylline against placebo, in non-intubated children, on the subsequent need for mechanical ventilation.

#### 3) Mortality

There were no reported deaths in any study.

### Secondary outcomes

#### 1) Date until discharge criteria are met

One study reported time until children were ready for discharge home and found no significant difference between theophylline and placebo (27.0±10.3 hours vs 30.4±16.8 hours [p>0.05]. Mean theophylline level 13.1mg/l) [16]. Another study measured time to meet discharge criteria from the intensive care unit, but not time until discharge home. The study reported a statistically significant difference in favour of aminophylline (29.8±4.9h vs 36.4±5.5h [p<0.05]. Mean daily theophylline level = 14.5 ± 0.7 mg/L, target theophylline levels 12-17mg/l) [18].

#### 2) Actual discharge

Four studies recorded length of time in hospital as an outcome. One study, in which mean theophylline levels were 7.2mg/l [15] and one study with mean levels of 12.3mg/l [20] demonstrated no statistically significant difference in length of hospital stay when compared to placebo. One trial demonstrated a significant improvement in length of stay in critical care in the aminophylline group compared with placebo (3.9±0.3 days versus 8.8±1.5 days in placebo [p<0.05] mean serum theophylline level 11.2mg/l), but not in discharge home (8.3±1.5 days versus 13.0±1.0 days [p>0.05] mean serum theophylline level 11.2mg/l). This study demonstrated a significantly shorter length of stay in critical care in the very small subset of intubated patients receiving aminophylline compared to those receiving placebo [18].

One retrospective study found that length of stay in critical care was longer for subjects receiving aminophylline (hazard ratio 0.396, [p = 0.001], 63% of participants >10mg/l) but does not follow up patients until discharge home. Of those receiving aminophylline, those found to have levels >10mg/l were found to have a longer stay in the intensive care unit compared to those who with levels <10 mg/l [24]. Another retrospective study reported the mean length of stay of hospital for patients receiving aminophylline was 3.25 days, but no comparison is made between those achieving different serum theophylline levels [25].

#### 3) Adverse effects

Eight RCTs reported adverse effects. Three studies demonstrate statistically significantly higher rates of adverse effects in those receiving intravenous theophyllines compared to placebo [20–22] whilst an other study demonstrates no significant difference [19]. In the few research participants with supratherapeutic theophylline levels (>20 mg/l) there did not appear to be an increased risk of side effects. One retrospective study reported no adverse effects in any of its supratherapeutic patients [25]. and one study links adverse effects to an individual participant who experienced nausea and abdominal pain with levels of 23mg/l [22].

#### 4) Spirometry

Three studies reported FEV_1_ as an outcome. Two studies demonstrated significant improvements in FEV_1_ in the theophylline group compared to placebo (22.5% vs 13.1% [p = 0.029], serum level of participants 10-20mg/l) [20] (89% vs 62% [p<0.001], serum level of participants 5-15mg/l) [17] whilst another study with theophylline levels between 10-20mg/l demonstrated no statistically significant difference in FEV_1_,[23] In other studies, a large proportion of participants were unable or unwilling to perform spirometry

## Discussion

There is no evidence to suggest that 10–20 mg/l of theophylline is the optimal target serum range in children with severe acute asthma. Across studies comparing aminophylline to placebo there appears to be no difference in outcome between concentrations of 10–20 mg/l and <10mg/l. There is weak evidence to suggest that levels >20 mg/l are associated with in increase in abdominal pain, nausea and vomiting. There is insufficient evidence to suggest that higher serum levels could result in an improvement of symptoms.

This review demonstrates that there is an unclear relationship between serum levels and either clinical efficacy or development of adverse effects. For evidence based therapeutic range to be determined, there is a need for RCTs comparing ranges and measuring important clinical outcomes, to determine the optimal dose in children. Until there is clear evidence that the beneficial serum level of theophylline lies within a certain range, rigorous evaluation of clinical progress and adverse drug effects should be used to guide therapy rather than laboratory investigations.

Data suggests that a 5mg/kg loading dose would leave one third of children would be below 10mg/l, and none above 20mg/l [27] Routine measurement of serum theophylline levels in children suffering acute asthma who have received standard loading doses of aminophylline to achieve serum concentrations in the 10-20mg/L range is therefore unlikely to result in any clinical benefit or reduction in adverse effects. However measurement of serum theophylline in childhood acute severe asthma may still retain utility in the assessment of patients in whom there is concern about overdose.

As we were unable to identify any RCTs directly comparing target ranges of theophylline, our analyses incorporate indirect observational comparison across studies.

This review was hindered by inconsistencies between studies in measurement and reporting of serum theophylline levels, and poor measurement of outcomes that are consistent with up to date research investigating clinically relevant outcomes in childhood asthma [11]. Furthermore, our included studies span a 43 year time period and changes clinical practice, administration of IV aminophylline and the selection of outcomes present further challenges when comparing results. All of these issues contribute to data heterogeneity. Meta analysis was considered but is unlikely to provide further insight into the optimum therapeutic range of aminophylline.

Research in children presents specific challenges such as potential difficulty in reporting subjective side effects and reluctance to take blood samples, so monitoring of adverse effects may be difficult. We agree with the a need for consistent reporting of adverse effects in clinical trials [13]. A core outcome set is needed to measure and report outcomes in all trials. This should be developed using rigorous consensus methodology [28] and would help interpretation of studies, enable synthesis across trials, and reduce reporting bias [29].

## Conclusion

There is no evidence that theophylline levels above 10mg/l compared with levels below 10mg/l are associated with improvement in children with severe acute asthma, nor that levels below 20mg/l are associated with less adverse effects than higher levels. Even if theophylline levels are measured, we recommend that clinicians should be guided by clinical improvement, and be vigilant to adverse effects, rather than simply titrate the dose according to serum levels. High quality RCTs are required to compare therapeutic ranges of intravenous theophylline in children, and these should measure and report a standardized core set of validated outcome measures reflecting both benefits and harms.

## Supporting Information

S1 File PRISMA 2009 Checklist. (DOC)

S2 File Excluded studies after reading full text. (DOCX)

## References

[1] WHO. Asthma factsheet [internet]. Nov 2013. [Cited Nov 2015]. available from: http://www.who.int/mediacentre/factsheets/fs307/en/.

[2] Kenyon N, Zeki AA, Albertson TE, Louie S. Definition of critical asthma syndromes. Clin Rev Allergy Immunol. 2015;48(1): 1–6. 10.1007/s12016-013-8395-6 24213844 PMC6063533

[3] British Thoracic Soceity. SIGN 141 british guideline on the management of asthma [internet]. 2014. [Cited Nov 2015]. available from: https://www.brit-thoracic.org.uk/document-library/clinical-information/asthma/btssign-asthma-guideline-2014/.

[4] Barnes PJ. Theophylline. Am J Respir Crit Care Med. 2013;188(8):901–6. 10.1164/rccm.201302-0388PP 23672674

[5] Resar RK, Walson PD, Fritz WL, Perry DF, Barbee RA. Kinetics of theophylline; variability and effect of arterial ph in chronic obstructive lung disease. CHEST Journal. 1979;76(1):11–6. 10.1378/chest.76.1.11 36261

[6] Soldin OP, Soldin SJ. Review: Therapeutic drug monitoring in pediatrics. Ther Drug Monit. 2002;24(1):1–8. 11805714 10.1097/00007691-200202000-00001 PMC3641772

[7] Paediatric Formulary Committee. British national formulary for children. London: BMJ Group and Pharmaceutical Press; 2014 141p.

[8] Turner-Warwick M. Study of theophylline plasma levels after oral administration of new theophylline compounds. Br Med J. 1957;2(5036):67–9. 13436856 10.1136/bmj.2.5036.67 PMC1961780

[9] Jackson RH, McHenry JI, Moreland FB, Raymer WJ, Etter RL. Clinical evaluation of elixophyllin with correlation of pulmonary function studies and theophylline serum levels in acute and chronic asthmatic patients. CHEST Journal. 1964;45(1):75–85. 10.1378/chest.45.1.75 14114639

[10] Mitenko PA, Ogilvie RI. Rational intravenous doses of theophylline. N Engl J Med. 1973;289(12):600–3. 4723589 10.1056/NEJM197309202891202

[11] Sinha IP, Gallagher R, Williamson PR, Smyth RL. Development of a core outcome set for clinical trials in childhood asthma: A survey of clinicians, parents, and young people. Trials. 2012;13(1):103. 22747787 10.1186/1745-6215-13-103 PMC3433381

[12] Higgins JPT, Altman DG, Gøtzsche PC, Jüni P, Moher D, Oxman AD, et al The cochrane collaboration’s tool for assessing risk of bias in randomised trials. BMJ: British Medical Journal. 2011;343:d5928 10.1136/bmj.d5928 22008217 PMC3196245

[13] Loke YK, Price D, Herxheimer A. Systematic reviews of adverse effects: Framework for a structured approach. BMC Medical Research Methodology. 2007;7(1):32. 17615054 10.1186/1471-2288-7-32 PMC1933432

[14] Needleman JP, Kaifer MC, Nold JT, Shuster PE, Redding MM, Gladstein J. Theophylline does not shorten hospital stay for children admitted for asthma. Archives of Pediatrics and Adolescent Medicine. 1995;149(2):206–9. 7849886 10.1001/archpedi.1995.02170140088016

[15] Silveira D'Ávila R, Piva JP, José Cauduro Marostica P, Luís Amantea S. Early administration of two intravenous bolus of aminophylline added to the standard treatment of children with acute asthma. Respiratory Medicine. 2008;102(1):156–61. 17869497 10.1016/j.rmed.2007.07.030

[16] DiGiulio GA, Kercsmar CM, Krug SE, Alpert SE, Marx CM. Hospital treatment of asthma: Lack of benefit from theophylline given in addition to nebulized albuterol and intravenously administered corticosteroid. J Pediatr. 1993;122(3):464–9. 8441107 10.1016/s0022-3476(05)83442-0

[17] Pierson W, Bierman W, Stamm S. Double-Blind trial of aminophylline in status asthmaticus. Pediatrics. 1971;48(4):642 4940006

[18] Ream RS, Loftis LL, Albers GM, Becker BA, Lynch RE, Mink RB. Efficacy of IV theophylline in children with severe status asthmaticus. Chest. 2001;119(5):1480–8. 11348957 10.1378/chest.119.5.1480

[19] Nuhoğlu Y, Dai A, Barlan IB, Başaran MM. Efficacy of aminophylline in the treatment of acute asthma exacerbation in children. Annals of Allergy, Asthma & Immunology: Official Publication of the American College of Allergy, Asthma, & Immunology. 1998;80(5):395–8. 10.1016/S1081-1206(10)62990-0 9609609

[20] Yung M, South M. Randomised controlled trial of aminophylline for severe acute asthma. Archives of Disease in Childhood. 1998;79(5):405–10. 10193252 10.1136/adc.79.5.405 PMC1717748

[21] Bien JP, Bloom MD, Evans RL, Specker B, O'Brien KP. Intravenous theophylline in pediatric status asthmaticus: A prospective, randomized, double-blind, placebo-controlled trial. Clinical Pediatrics.1995;34(9):475–81. 7586920 10.1177/000992289503400905

[22] Strauss RE, Wertheim DL, Bonagura VR, Valacer DJ. Aminophylline therapy does not improve outcome and increases adverse effects in children hospitalized with acute asthmatic exacerbations. Pediatrics. 1994;93(2):205–10. 8121733

[23] Carter E, Cruz M, Chesrown S, Shieh G, Reilly K, Hendeles L. Efficacy of intravenously administered theophylline in children hospitalized with severe asthma. J Pediatr. 1993;122(3):470–6. 8441108 10.1016/s0022-3476(05)83443-2

[24] Dalabih AR, Bondi SA, Harris ZL, Saville BR, Wang W, Arnold DH. Aminophylline infusion for status asthmaticus in the pediatric critical care unit setting is independently associated with increased length of stay and time for symptom improvement. Pulmonary Pharmacology and Therapeutics. 2014;27(1):57–61. 10.1016/j.pupt.2013.03.001 23523660 PMC3732550

[25] Fox J, Hicks P, Feldman BR, Davis WJ. Theophylline blood levels as a guide to intravenous therapy in children. American Journal of Diseases of Children. 1982;136(10):928–30. 7124680 10.1001/archpedi.1982.03970460058012

[26] DiGiulio GA, Kercsmar CM, Krug SE, Alpert SE, Marx CM. Hospital treatment of asthma: Lack of benefit from theophylline given in addition to nebulized albuterol and intravenously administered corticosteroid. J Pediatr. 1993;122(3):464–9. 8441107 10.1016/s0022-3476(05)83442-0

[27] Gleeson JG, Price JF. Aminophylline dosage in acute severe asthma. Eur J Pediatr. 1989;148(6):577–8. 2744023 10.1007/BF00441563

[28] Sinha IP, Smyth RL, Williamson PR. Using the delphi technique to determine which outcomes to measure in clinical trials: Recommendations for the future based on a systematic review of existing studies. PLOS Med. 2011;8(1):e1000393 10.1371/journal.pmed.1000393 21283604 PMC3026691

[29] Sinha I, Jones L, Smyth RL, Williamson PR. A systematic review of studies that aim to determine which outcomes to measure in clinical trials in children. PLOS Med. 2008;29;5(4):e96 10.1371/journal.pmed.0050096 18447577 PMC2346505

