# Supplementary material for: The Evidence for Intravenous Theophylline Levels between 10-20mg/L in Children Suffering an Acute Exacerbation of Asthma: A Systematic Review
Source: PLoS One. 2016 Apr 20;11(4):e0153877. doi: 10.1371/journal.pone.0153877 (PMC4838302; doi:10.1371/journal.pone.0153877)
Supplement: S2 File — (DOCX) [file pone.0153877.s002.docx]

**S2 File. Excluded studies after reading full text**

| **Study** | **Reason** |
| --- | --- |
| Singhi 2014 [30] | Did not measure theophylline levels |
| Zainudin 1994 [31] | Includes adults and children, children not treated separately in results and statistical analysis |
| Ibrahim 1993 [32] | Did not measure theophylline levels |
| Shilalukey 1993 [33] | Did not measure any of the stated primary outcomes |
| Bowler 1987 [34] | Adult study |
| Carrier 1985 [35] | Adult study |
| Katz 1981 [36] | Includes adults and children, children not treated separately in results and statistical analysis |
| Blumenthal 1979 [37] | Correspondence article |
| Josephson 1979 [38] | Includes adults and children, children not treated separately in results and statistical analysis |
| Hambleton 1979 [39] | Did not measure theophylline levels |

31. Singhi S, Grover S, Bansal A, Chopra K. Randomised comparison of intravenous magnesium sulphate, terbutaline and aminophylline for children with acute severe asthma. International Journal of Paediatrics 2014;103(12):1301-6.

31. Zainudin BM, Ismail O, Yusoff K. Effect of adding aminophylline infusion to nebulised salbutamol in severe acute asthma. Thorax 1994; 49(3): 267-9.

32. Ibrahim SA, Elgurashi ED, Elkarim OA. Comparative study of intravenous aminophylline subcutaneous adrenaline and nebulised salbutamol in the treatment of acute asthma in children. Pediatric reviews and communications. 1993; 7(3): 175-82.

33. Shilalukey K, Robieux I, Spino M, Greenwald M, Shear N, Koren G. Are current pediatric dose recommendations for intravenous theophylline appropriate? J Asthma 1993; 30(2): 109-21.

34. Bowler SD, Mitchell CA, Armstrong JG, Scicchitano R. Nebulized fenoterol and i.V. Aminophylline in acute severe asthma. European Journal of Respiratory Diseases 1987; 70(5): 280-3.

35. Carrier JA, Shaw RA, Porter RS, Allison EJ, Kessler ER, Woody DG, et al. Comparison of intravenous and oral routes of theophylline loading in acute asthma. Ann Emerg Med 1985; 14(12): 1145-51.

36. Katz G, Kewitz G, Vozeh S, Follath F. Relationship between dose, serum concentration and side effects in intravenous aminophylline therapy. 1981; 111(52): 2054-6.

37. Blumenthal I, Tormey WP. Comparison of IV salbutamol with IV aminophylline in severe acute asthma. 1979; 54(12): 983-.

38. Josephson GW, MacKenzie EJ, Lietman PS, Gibson G. Emergency treatment of asthma. A comparison of two treatment regimens. 1979; 242(7): 639-43.

39. Hambleton G, Stone MJ. Comparison of IV salbutamol with IV aminophylline in the treatment of severe, acute asthma in childhood. Archives of Disease in Childhood 1979; 54(5): 391-2.
